# Spherical particles of halophilic archaea correlate with exposure to low water activity – implications for microbial survival in fluid inclusions of ancient halite

**DOI:** 10.1111/j.1472-4669.2012.00337.x

**Published:** 2012-07-15

**Authors:** S Fendrihan, M Dornmayr-Pfaffenhuemer, F W Gerbl, A Holzinger, M Grösbacher, P Briza, A Erler, C Gruber, K Plätzer, H Stan-Lotter

**Affiliations:** 1 Romanian Bioresource Centre and Advanced Research AssociationBucharest, Romania; 2 Division of Molecular Biology, University of SalzburgSalzburg, Austria; 3 Institute of Groundwater Ecology, Helmholtz Zentrum München – German Research Center for Environmental HealthNeuherberg, Germany; 4 Division of Molecular Biology, University of SalzburgSalzburg, Austria; 5 Division of Cell Biology, University of SalzburgSalzburg, Austria; 6 Division of Material Science and Physics, University of SalzburgSalzburg, Austria

## Abstract

Viable extremely halophilic archaea (haloarchaea) have been isolated from million-year-old salt deposits around the world; however, an explanation of their supposed longevity remains a fundamental challenge. Recently small roundish particles in fluid inclusions of 22 000- to 34 000-year-old halite were identified as haloarchaea capable of proliferation (Schubert BA, Lowenstein TK, Timofeeff MN, Parker MA, 2010, *Environmental Microbiology,* 12, 440–454). Searching for a method to produce such particles in the laboratory, we exposed rod-shaped cells of *Halobacterium* species to reduced external water activity (a_w_). Gradual formation of spheres of about 0.4 μm diameter occurred in 4 m NaCl buffer of a_w_ ≤ 0.75, but exposure to buffered 4 m LiCl (a_w_ ≤ 0.73) split cells into spheres within seconds, with concomitant release of several proteins. From one rod, three or four spheres emerged, which re-grew to normal rods in nutrient media. Biochemical properties of rods and spheres were similar, except for a markedly reduced ATP content (about 50-fold) and an increased lag phase of spheres, as is known from dormant bacteria. The presence of viable particles of similar sizes in ancient fluid inclusions suggested that spheres might represent dormant states of haloarchaea. The easy production of spheres by lowering a_w_ should facilitate their investigation and could help to understand the mechanisms for microbial survival over geological times.

## Introduction

During the past years, renewed interest in the presence of viable prokaryotes in ancient salt deposits has surfaced, which was partly stimulated by the detection of halite on Mars (Treiman *et al*., 2000; Squyres *et al*., 2006) and elsewhere in the universe (Zolensky *et al*., 1999). The first cultivations of halophilic micro-organisms from Permian salt deposits (about 250 million years old) were reported in the 1960s (Reiser & Tasch, 1960; Dombrowski, 1963) and met with considerable skepticism. Some 30 years later, successful isolations of halophilic bacteria and archaea (haloarchaea) from ancient evaporites, including detailed taxonomic descriptions, began to be published (Norton *et al*., 1993; Denner *et al*., 1994; Stan-Lotter *et al*., 2002; Mormile *et al*., 2003; Gruber *et al*., 2004; Vreeland *et al*., 2007). No methods are available yet which would be sensitive enough to ascertain the age of a single prokaryotic cell. The age of the salt sediments, which can be estimated by radioactive dating, stratigraphy, and pollen analysis (Stan-Lotter *et al*., 2004; and references therein), is therefore deemed the presumed age of the micro-organisms found inside. This perception has raised many discussions, mainly on the possibility of microbial contamination during isolation and processing of samples, as well as uncertainty about their real age (McGenity *et al*., 2000; Park *et al*., 2009; Schubert *et al*., 2010). Retrieval of DNA from ancient sediments (Radax *et al*., 2001; Fish *et al*., 2002), repeated isolations from the same site (Stan-Lotter *et al*., 1999), and recent reports of successful haloarchaeal cultivations from well-dated bore cores (Schubert *et al*., 2010; Gramain *et al*., 2011) supported the presence of biological material in evaporites of great geological age. Survival of cells over millenia of years in dry sediments or sedimentary rocks on Earth would have important implications for the search for life on other planets, where sediment-like structures exist (Squyres & Knoll, 2005).

Plausible mechanisms for the long-term survival of extremely halophilic archaea in salt sediments have yet to be clarified. Genome sequences suggested that they do not produce spores (Onyenwoke *et al*., 2004), which are resting states of some bacteria and eukaryotes for survival of unfavorable conditions. The occurrence of other types of haloarchaeal resting states, such as cysts, has been discussed (Grant *et al*., 1998), but could not yet be demonstrated unequivocally. Fluid inclusions in halite were considered early as possible habitats for micro-organisms (Norton & Grant, 1988). Indeed, a strain of *Halobacterium salinarum* was isolated from a single fluid inclusion in a 97 000-year-old halite crystal from Death Valley (Mormile *et al*., 2003). Recently, the presence of small spherical particles with <1 μm diameter in fluid inclusions of modern and ancient halite was reported (Schubert *et al*., 2009). The occurrence of roundish cells in laboratory-grown halite had also been described previously (Norton & Grant, 1988). Importantly, the successful propagation of spherical particles, which had been observed microscopically in fluid inclusions of 22 000- to 34 000-year-old halite, was demonstrated (Schubert *et al*., 2010); the resulting cultures were identified as three different genera of haloarchaea. Improved visualization by staining cells with fluorescent dyes had earlier revealed the preferential accumulation of haloarchaea (Fendrihan & Stan-Lotter, 2004; Fendrihan *et al*., 2006) and bacteria (Adamski *et al*., 2006) within fluid inclusions of laboratory-grown halite and not in the host crystal matrix. After prolonged entrapment, rod-shaped species of *Halobacterium* were apparently converted to roundish particles (Fendrihan & Stan-Lotter, 2004). In the absence of spore formation, it is tempting to assume that the spheres in ancient fluid inclusions represent a form of haloarchaeal resting state. Most haloarchaea possess an S-layer (surface layer), which forms the outer envelope of the cell and consists of glycoproteins, held together by non-covalent interactions (see Sára & Sleytr, 2000; for a review). They do not contain the covalently linked rigid peptidoglycan, which constitutes the cell wall of many bacteria. The haloarchaeal S-layer can be removed by chelating agents, leaving large fragile spheroplasts (Cline & Doolittle, 1992). Nothing is known yet about the mode of formation and properties of small haloarchaeal spheres, although some early reports described spheres of various sizes and stabilities when exploring the effects of different salts on halophilic archaea (Abram & Gibbons, 1961; Kushner & Bayley, 1963; Mohr & Larsen, 1963). Here we show that sphere formation is apparently a response to low external water activity (a_w_ or aH_2_O) of several haloarchaeal species. We also describe a surprisingly simple and rapid procedure for the preparation of viable spheres, which should be useful for further molecular and biophysical studies to elucidate the long-term survival of micro-organisms in sediments.

## Material and methods

### Haloarchaeal strains and culture conditions

Strains *Halobacterium salinarum* DSM 670, *Hbt. salinarum* DSM 3754^T^, *Hbt. noricense* DSM 15987^T^, and *Haloferax mediterranei* DSM 1411^T^ were obtained from the Deutsche Sammlung von Mikroorganismen und Zellkulturen (DSMZ), Braunschweig, Germany. Strain *Hbt. salinarum* NRC-1 (ATCC-700922) was purchased from LGC Teddington (Teddington, UK). *Halobacterium* strains DSM 670, DSM 3754^T^, and DSM 15987^T^ were grown in DSM 823 medium (http://www.dsmz.de/microorganisms/medium/pdf/DSMZ_Medium823.pdf). *Hfx. mediterranei* DSM 1411^T^ was grown in DSM 954 medium (http://www.dsmz.de/microorganisms/medium/pdf/DSMZ_Medium954.pdf). *Hbt. salinarum* NRC-1 was grown in ATCC 2185 medium (http://www.lgcstandards-atcc.org/Attachments/3339.pdf). The pH of the growth media was 7.0. For solid media, 20 g L^−1^ agar was added. Incubation of cultures was at 37 °C. Growth in liquid culture was monitored at 600 nm with a spectrophotometer (Novaspec II, Pharmacia, Vienna, Austria).

### Embedding of haloarchaea in salt crystals

When the cultures reached an optical density (OD) of 0.9–1.3 [equal to approximately 10^8^ to 5 × 10^9^ colony-forming units (CFU) per mL], cells were harvested by centrifugation at 7000 *g* for 20 min at 20 °C (Sorvall RC6, rotor SLA 3000). The pellets were washed by resuspending them at a ratio of 500 mg (wet weight) per 10 mL of TN buffer (100 mm Tris–HCl, 4 m NaCl, pH 7.4, adjusted with HCl) and centrifuged as before. Five milliliters each of the suspension was placed into 94-mm diameter petri dishes and stored in an incubation chamber at 37 °C, unless noted otherwise, for up to 70 days. The weight of the petri dishes was determined gravimetrically; after about 5–6 days of incubation, it did not decrease any further. For small-scale preparations, 50 μL of a haloarchaeal cell suspension, which was stained with the LIVE/DEAD® *Bac*Light™ bacterial viability kit (Invitrogen, Lofer, Austria) as described previously (Leuko *et al*., 2004), was dried onto a glass slide for 2–3 days at ambient temperature in the dark.

### Preparation of haloarchaeal spheres

Following embedding in halite for several days, salt crystals were dissolved by the addition of modified TN buffer that contained 3 m NaCl (instead of 4 m). If required, concentration of samples was performed by centrifugation at 10 000 *g* in a 4K-15 centrifuge (Sigma, Vienna, Austria) at 20 °C for 15–20 min. In later experiments, spheres were prepared by suspending haloarchaeal pellets in TL buffer, which consisted of 100 mm Tris–HCl, 4 m LiCl, pH 7.4 (adjusted with HCl) at a ratio of about 50 mg of cells (wet weight) per ml of buffer. Suspensions were left for several hours or overnight at ambient temperature. The resulting spheres were centrifuged as noted above and washed twice with TN buffer to remove LiCl.

### Electron and light microscopy

Cells or spheres were prepared for scanning electron microscopy (SEM) by fixing with 4% glutaraldehyde in 0.15 m cacodylate buffer, containing 4.7 m NaCl, 80 mm MgSO_4_ × 7H_2_O, post-fixing with 1% osmium tetroxide, critical point-drying, and sputter-coating with approximately 2 nm Pt. A Hitachi S-900 field emission SEM was used. This work was carried out by Chris Frethem at the University of Minnesota Characterization Facility. Dimensions of cells and spheres were determined from electron micrographs. Five measurements were made for cells (rods) and 21 measurements for spheres.

Fluorescence microscopy of cells stained with the LIVE/DEAD® *Bac*Light™ bacterial viability kit, referred to as LIVE/DEAD kit, was carried out as described previously (Leuko *et al*., 2004). For some experiments, a laser confocal microscope (Zeiss KLM 510, Vienna, Austria), equipped with an argon laser for excitation at 488 nm, was used. Photographs were taken with single-track two-channel examination, which allows mixing of images. The instrument was operated by the dedicated software Zeiss LSM 5, version 3.2.

Unstained cells were observed with the Leica (Vienna, Austria) DM 5000B microscope or an Eclipse E200 microscope (Nikon, Vienna, Austria), using phase contrast. Photography of light microscopic images was performed with a Powershot G10 camera (Canon, Vienna, Austria). For quantitative determinations, aliquots of cells and spheres were examined in a Thoma microscopic counting chamber (Lactan, Graz, Austria). Cultures with 3 × 10^8^ CFU were used. Ratios of spheres to cells were determined with cultures from mid-log, late-log, and late stationary phase. At least 10 determinations were done for each growth phase.

### Biochemical and physiological tests

Lysis of haloarchaea in water was determined by suspending cells or spheres in sterile distilled water and observing cellular integrity, or the loss of it, by light microscopy for 1–2 h. The enzymes cytochrome oxidase and catalase were tested by standard procedures (Smibert & Krieg, 1994). For additional enzyme activities, the Analytical Profile Index system API ZYM (bioMerieux, Vienna, Austria) was used (Humble *et al*., 1977). All API tests were performed at least three times.

Polar lipids were extracted, separated by thin-layer chromatography, and visualized as described (Stan-Lotter *et al*., 2002). The equivalence of spots was determined by cochromatography with the extract of *Halobacterium salinarum* NRC-1, whose polar lipid composition is known (Gruber *et al*., 2004).

### SDS polyacrylamide gel electrophoresis (SDS–PAGE)

SDS–PAGE was performed using the gel system by Laemmli (1970). For whole-cell proteins, approximately 50 mg mL^−1^ of cells or spheres (wet weight) was lysed by boiling in SDS sample buffer (Laemmli, 1970) for 10 min. For examination of released proteins, spheres that had been prepared by treatment with TL buffer were centrifuged at 10 000 *g* for 20 min. Supernatants were taken off and filtered through 0.22-μm sterile filters to remove any residual spheres. Filtered samples were applied to SDS polyacrylamide gels (criterion gradient gels 10.5–14% acrylamide; Bio-Rad, Vienna, Austria), following mixing with SDS sample buffer (Laemmli, 1970) in a ratio of 1:1, or, if required, were concentrated first about threefold with Centricon devices (Amicon) with a cutoff of 10 000 Da. Proteins were stained with Coomassie blue. Electrophoresis of whole-cell proteins and supernatants from spheres was repeated more than four times.

### Mass spectrometry of proteins

Sequence analysis of proteins that were released into the supernatant, following sphere preparation by treatment with 4 m LiCl, was performed by LC-MS/MS. Proteins were separated by SDS polyacrylamide gel electrophoresis as described earlier. Coomassie-stained bands 1–7 (see Fig. S1) were excised and in-gel digested using the ProteoExtract All-In-One Trypsin Digestion Kit (Calbiochem, San Diego, CA, USA). Resulting peptides were separated by RP-HPLC (Nanoease Symmetry 300™ trap column and Nanoease Atlantis dC18™ separating column; Waters, Milford, MA, USA) directly coupled to an ESI quadrupole TOF mass spectrometer (Q-Tof Ultima Global; Waters/Micromass). For sequence identification, a combined Swiss-Prot/TrEMBL database was used. Experimental details and instrument parameters used were described previously (Erler *et al*., 2011).

### Other methods

Colony-forming units were determined following plating of aliquots of exposed and resuspended cells or, in the case of liquid cultures, of cell suspensions, on agar plates of 90 mm diameter containing solidified DSM 954 medium or ATCC 2185 medium. Plates were incubated at 37–40 °C for several days. Determination of ATP was performed in a microassay method, using the luciferin–luciferase reaction in 96-well microplates (Kiesslich *et al*., 2003). Reagents were obtained from Sigma-Aldrich except for luciferin (Invitrogen, Vienna, Austria) and luciferase (Promega, Mannheim, Germany). Luminescence was measured in an Infinite M200 microplate reader (Tecan, Groedig, Austria). Determination of protein was by Lowry′s method (Lowry *et al*., 1951), with BSA as standard. The number of determinations was at least 5 in both experiments. Water activity (a_w_) was determined with the portable Pawkit (IUL Instruments GmbH, Königswinter, Germany), using salt standards of known a_w_, which were supplied by the company (LiCl 0.25 a_w_, NaCl 0.76 a_w_), for calibration.

An estimation of sphere size was made *in vivo* by passing sphere suspensions of *Hbt. salinarum* NRC-1 (ca. 10^9^ CFU mL^−1^) through filters of pore sizes 0.2, 0.45, 0.8, 1.2, and 5 μm (Minisart; VWR, Vienna, Austria), respectively, and examination of recovered cells in the filtrate by subsequent microscopy, using phase contrast. Suspensions of rod-shaped cells (ca. 10^8^ CFU mL^−1^) served as controls.

## Results

### Formation of spheres in fluid inclusions of halite

For slow embedding of haloarchaeal cells in halite, cultures of *Halobacterium* species in Tris-buffered 4 m NaCl (TN buffer) were left to dry at ambient temperature or 37 °C and stored for several days or weeks (see Material and Methods). On a smaller scale, drying was carried out on glass slides with haloarchaeal cells, which had been pre-stained with the LIVE/DEAD® *Bac*Light™ bacterial viability kit, referred to as LIVE/DEAD kit, before embedding. The bright green fluorescence of cells stained with the dye SYTO9 outlined the shapes of the characteristic square or rectangular fluid inclusions of halite (Fig., left). Upon higher magnification, spherical particles of *Halobacterium salinarum* NRC-1, entrapped within a fluid inclusion, became visible (Fig., right). Most cells were fluorescing green, indicating intact membranes and therefore viability. Embedding in halite evidently caused transformation of formerly rod-shaped cells to nearly uniformly spherical particles. Scanning electron microscopy of spheres obtained from *Hbt. salinarum* NRC-1, which were fixed with glutaraldehyde after dissolution of halite crystals (see Methods), showed mainly single globular particles (Fig.B,D,F) with a diameter of 0.40 ± 0.02 (SD) μm. The diameter of the rods of *Hbt. salinarum* NRC-1 was about 0.65 μm (Fig.A; Kushner & Bayley, 1963). Occasionally spheres were adhering to each other in groups of 2–4 (Fig.C,E,G,H); as these arrangements were observed frequently, the assemblies most likely represented intermediate stages in the transition from rods to spheres. Images from fluorescence microscopy following staining of cells with the LIVE/DEAD kit contained frequently groups of three spheres (Fig.), corroborating the notion that one cell gave rise to several spheres and that adhesion of spheres persisted for some time.

**Fig. 1:**
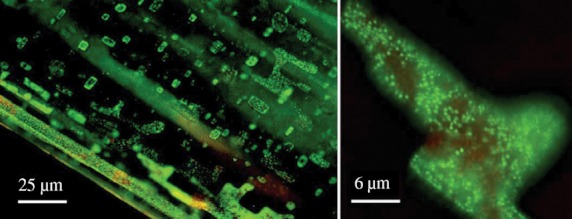
Localization of pre-stained cells of *Halobacterium salinarum* NRC-1 in halite fluid inclusions. Low magnification (left) and higher magnification of an individual fluid inclusion (right). Cells were stained with the LIVE/DEAD® *Bac*Light™ bacterial viability kit prior to embedding in halite; epifluorescence microscopy was performed after entrapment of cells for 3 days.

**Fig. 2:**
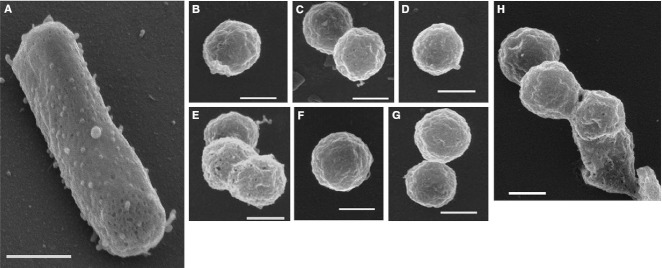
Scanning electron micrographs of rods (A) and spheres (B–H) of *Halobacterium salinarum* NRC-1. Spheres had formed in laboratory-grown halite and were obtained after dissolution of salt crystals. Bars, 600 nm (A); 270 nm (B–H).

**Fig. 3:**
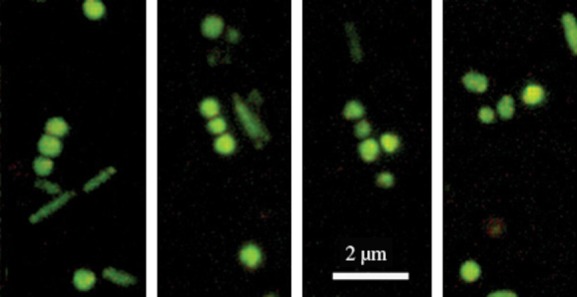
Groups of spheres from *Halobacterium salinarum* NRC-1, following embedding of rod-shaped cells in halite for 30 days, dissolution of salt crystals and staining with the LIVE/DEAD kit.

The water activity (a_w_) of halophilic growth medium as well as TN buffer was 0.75 at ambient temperature, measured with a capacitance hygrometer (Paw kit; see Material and Methods). Mere storage of haloarchaeal rods in TN buffer did not alter their morphology. Therefore, we concluded that the water activity within fluid inclusions presumably decreased below 0.75 upon prolonged evaporation and that this reduction caused the formation of spherical particles.

### Rapid conversion of rods to spheres by LiCl

If sphere formation is triggered by lowering of a_w_, other salt solutions may bring about this effect, too. Replacement of Na^+^ with other cations (K^+^, Mg^++^, Ca^++^) or mixtures of such salts resulted in partial conversion of rods into large and small spheres, similar as observed by Abram & Gibbons (1961); in addition, cells or spheres often lost viability. However, suspension of rod-shaped haloarchaea in Tris-buffered 4 m LiCl (TL buffer) of an a_w_ of 0.73 at 22–23 °C led to nearly immediate appearance of spheres (Fig.). The method was a convenient way to produce homogenous preparations of spheres. No influence of growth phase was noted, because cells in mid-log to late-log to stationary phase (with optical densities between 0.8 and 1.3) were always converted to spheres. The viability of haloarchaeal cells, when stored in TL buffer, declined within 2 days to near zero, as determined by CFU. Therefore, spheres made by exposure to 4 m LiCl were re-equilibrated in NaCl-containing buffer shortly after their formation (see Material and Methods).

**Fig. 4:**
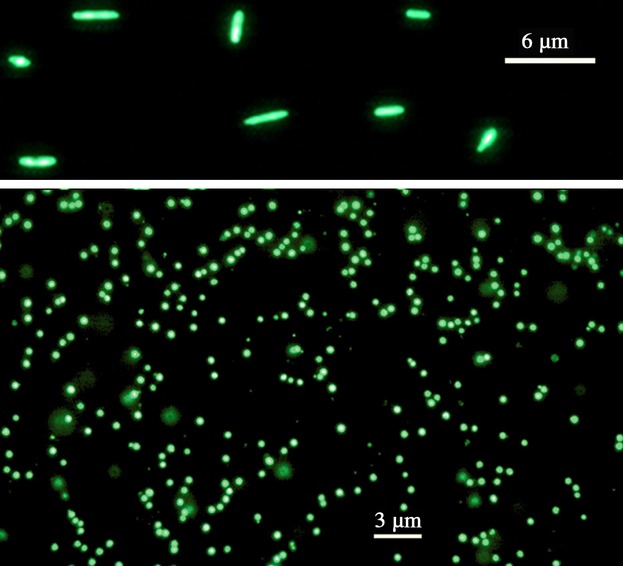
Rods (upper panel) and spheres (lower panel) of *Halobacterium salinarum* NRC-1, following staining with the LIVE/DEAD kit. These spheres were produced by exposure of rods to Tris-buffered 4 m LiCl.

The ratio of spheres to cells was 2.86 ± 0.97 (SD). This number was determined by using a microscopic counting chamber with cultures from mid-log, late-log, and stationary phase, respectively, which were counted before and after exposure to TL buffer. The ratio corroborated the previous observation that one rod gave rise to three or four spheres (see Figs 2 and 3).

### Properties of spheres

A phenotypical characterization of spheres in comparison with rods is shown in Table 1. Besides the obvious differences of their dimensions, rods and spheres differed in ATP content, which was about 50-fold lower in spheres, and a slower sedimentation of spheres. Whole-cell protein SDS gel electrophoresis showed a reduced amount of the S-layer protein in spheres (Fig.), which is a glycoprotein and forms the outermost coat of *Halobacterium* species (Mescher & Strominger, 1976) and several other haloarchaea. Its apparent molecular mass is about 200 kDa (see Fig.). Spheres contained the known polar lipids of *Hbt. salinarum* (Gruber *et al*., 2004; Table 1); however, the higher centrifugal force needed for sedimentation (Table 1) was probably due to a relative increase of lipids in spheres. The results suggested a loss of proteins, ATP, and probably also other molecules during the conversion from rods to spheres, which decreased the specific weight of spheres. The proteins, which were released upon conversion, were separated by SDS polyacrylamide gel electrophoresis (Fig. S1), and some of the more prominent ones were subsequently identified by mass spectrometry (Table 2). The S-layer protein of *Halobacterium* made up the bulk of released proteins; other proteins of smaller M_r_ were also present (Fig. S1; Table 2). Lag phases of growth in liquid and on solid medium were increased for spheres (Table 1); other properties of rods and spheres examined here were very similar.

**Fig. 5:**
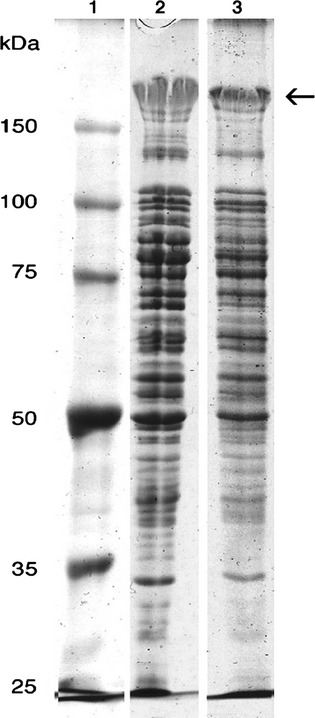
Whole cellular proteins of rods and spheres of *Halobacterium salinarum* NRC-1 following separation by SDS–PAGE. Acrylamide concentration was 12%. Approximately 25 μg of protein was loaded per lane. Lanes: 1, molecular mass markers; 2, *Hbt. salinarum* NRC-1, rods; 3, *Hbt. salinarum* NRC-1, spheres. Arrow (right) indicates position of the S-layer protein.

**Table 1:** Properties of spheres and rods from *Halobacterium salinarum* NRC-1

Property	Rods	Spheres
Shape	Cylindrical rods	Globule
Dimensions (from SEM)		
Length (μm)	2.5–4	–
Diameter (μm)	0.65 ± 0.06 (SD)	0.40 ± 0.02 (SD)
Lysis in distilled water	Yes	Yes
Main polar lipids	PGP-Me*	PGP-Me*
	PGS*, PG*	PGS*, PG*
	S-TGD-1*	S-TGD-1*
	S-TeGD*	S-TeGD*
Pigments (carotenoids)	Dark red	Light red
Enzymes assayed for		
Alkaline phosphatase	+	+
Alpha chymotrypsin	+	+
Acid phosphatase	+	+
Naphtol AS Bi phosphohydrolase	+	−
Oxidase	+	+
Catalase	+	+
ATP content (ng per mg protein)	620.0 ± 85 (SD)	12.4 ± 15 (SD)
Sedimentation at	2500–3200 *g*	8000–10 000 *g*
Lag phase (after dilution of 1:50 in fresh medium)	1–2 days	10–12 days
Lag phase (on agar plates)	4 days	≥10 days

Data for rods are from Gruber *et al*. (2004), except for ATP, which was determined in this work. For experimental details, see Material and Methods.

* PGP-Me, phosphatidyl glycerol phosphate methylester; PGS, phosphatidyl glycerol sulfate; PG, phosphatidyl glycerol; S-TGD-1, sulfated triglycosyl diether; S-TeGD, sulfated tetraglycosyl diether.

**Table 2:** Identification of *Halobacterium salinarum* DSM 670 proteins that were released during conversion from rods to spheres by exposure to TL buffer (Tris-buffered 4 m LiCl, pH 7.4), see Fig. S1

Band	UniProtKB accession number	Number of peptides identified	Sequence coverage (%)	PLGS score	M_r_ (kDa) theor./expt.	Function/name
1	CSG_HALSA	15	31.2	13.2	89.76*/200	Cell surface glyco-protein (S-layer)
2	CSG_HALSA	18	31.7	13.2	86.76*/185	Cell surface glyco-protein (S-layer)
3	Q9HMW9_HALSA	18	39.5	14.8	72.28/78	Dipeptide ABC transporter, dipeptide binding
4	Q9HMI3_HALSA	8	20.1	14.0	61.88/65	Dipeptide ABC transporter, ATP binding
5	Q9HPQ4_HALSA	8	24.0	14.2	49.44/61	Putative uncharac- terized protein
6	Q9HMU8_HALSA	7	24.0	14.2	31.85/42	Copper transport ATP binding protein
7	DPS_HALSA	5	57.0	13.2	20.10/28	DNA protection during starvation protein/Ferritin

Theoretical M_r_ were calculated using the Expasy server (http://web.expasy.org/cgi-bin/compute_pi/pi_tool).

* Sugar residues were not included.

The size of spheres as determined from SEM micrographs (Fig.; Table 1) was obtained after fixing and drying. An estimation of sphere size was also made *in vivo* by passing sphere suspensions of *Hbt. salinarum* NRC-1 through filters of pore sizes in the range between 0.2 and 5 μm (see Material and Methods). A cutoff at 0.45 μm was observed for spheres, corroborating the diameter of spheres as ≤0.45 μm.

Spheres, which were produced in laboratory-grown halite or by LiCl exposure, remained viable for at least 6 and 2 years, respectively, when stored in TN buffer. They preserved their morphology during this time and repeated staining with the LIVE/DEAD kit revealed green fluorescence, indicating viability. Upon streaking on agar plates, spheres produced normal colonies, which confirmed the preservation of propagation.

### Several haloarchaeal genera can form spheres

The data reported here were obtained with the closely related strains *Hbt. salinarum* NRC-1 and *Hbt. salinarum* DSM 670. We examined several other haloarchaeal species for the ability of sphere formation under reduced a_w_. Under similar conditions as described earlier, cultures of *Hbt. salinarum* DSM 3754^T^ and *Hbt. noricense* DSM 15987^T^ were exposed to buffered 4 m LiCl and found to be converted to spheres (Fig.). The pleomorphic haloarchaeon *Haloferax mediterranei* DSM 1411^T^ also produced spheres when exposed to 4 m LiCl (data not shown). These results suggested that the capacity for splitting cells into spheres at a_w_ below 0.75 is present in several genera of haloarchaea.

**Fig. 6:**
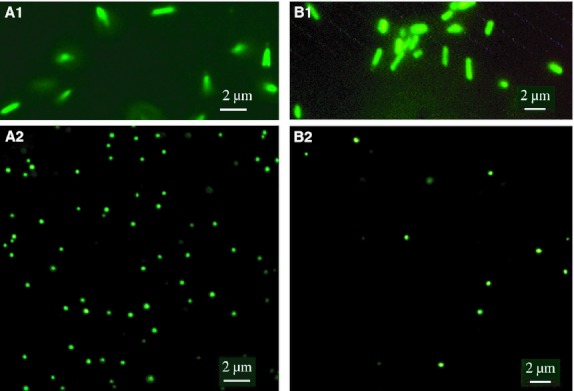
Rods (A1, B1) and spheres (A2, B2) of *Halobacterium salinarum* DSM 3854^T^ (A1, A2) and *Halobacterium noricense* DSM 15978^T^ (B1, B2), following staining with the LIVE/DEAD kit. Spheres were produced by exposure to Tris-buffered 4 m LiCl.

## Discussion

### Formation and viability of spheres

Our results suggested that rod-shaped cells of *Halobacterium* species gradually convert to small spheres upon a decrease of the external a_w_ to <0.75, as occurs in fluid inclusions of halite during evaporation. In primary halite fluid inclusions, values of a_w_ < 0.75 have indeed been measured (Yang *et al*., 1995). We showed that also in a liquid medium, outside of halite fluid inclusions, formation of haloarchaeal spheres can be induced, because upon encountering an environment with a_w_ ≪ 0.75, cells of *Halobacterium* split into spheres nearly instantaneously, when they were exposed to 4 m LiCl solutions of a_w_ ≤ 0.73. Spheres prepared either way – by evaporation of halite or addition of LiCl solution – showed identical biochemical properties (Table 1). The morphological shift to spheres was accompanied by release of a portion of the S-layer protein and several other proteins and molecules. The spheres remained stable and viable in NaCl-containing buffer of a_w_ of 0.75 under laboratory conditions for at least 6 years. Native cells of haloarchaea survive also under similar conditions, because water samples from the Dead Sea, which were stored for 56 years, contained numerous viable haloarchaeal species (Arahal *et al*., 2000), and *Halobacterium* species, which were entrapped in halite crystals for 27 months, could be re-grown (Gramain *et al*., 2011), albeit with extended times for recovery, as was observed here (longer lag phases; see Table 1. Haloarchaeal spheres formed normal rods (similar as shown in Fig. 2A and Figs 4 and 6, upper panels) when nutrients were available and propagation commenced. Obviously, the spheres contained complete genomes and probably multiple copies of them, because up to thirty genome copies can be present in growing cells of *Halobacterium salinarum* (Breuert *et al*., 2006). The mode of rapid breaking into spheres at low a_w_ suggested that preformed sites may exist in the cells, which allow fast splitting into several viable entities. The exact role of low a_w_ in this process is unknown; however, the speed of the conversion upon exposure to lithium ions would be compatible with conformational changes of proteins, perhaps caused by the removal of water shells, which triggers the fission of the cell. In any case, the mechanism constitutes an unknown mode of multiple cellular fission, perhaps with similarity to the type of proliferation which has been described for some bacteria and organelles (Angert, 2005), but not yet for archaea. The identification of proteins that were released upon formation of spheres indicated that the bulk consisted of the S-layer protein; in addition, several membrane proteins (Table 2, bands 3, 4 and 6) and one cytoplasmic protein (Table 2, band 7) were also released. The total surface area of three to four spheres of 0.4 μm diameter each would be much less than the surface area of an average haloarchaeal rod of about 0.65 × 2.5 μm (see Table 1); therefore, the release of a portion of the S-layer protein appears reasonable. A reduced amount of proteins, but retainment of other molecules, such as lipids, would be in agreement with the observed lower density of the spheres, compared to rods (Table 1). However, the analysis of released proteins given here should be considered as a preliminary result, which needs to be extended for a more meaningful interpretation, but it could hold the promise of elucidating the mechanism of multiple fission (Angert, 2005).

As mentioned earlier, transformations from rods to spheres of *Hbt. salinarum* had been reported in early studies of haloarchaea, caused by variation of pH, anions, and cations (Abram & Gibbons, 1961; Kushner & Bayley, 1963; Mohr & Larsen, 1963). In these cases, however, one cell was generally transformed into one large sphere, with a greater diameter than the rod-shaped progenitor and with a propensity to lysis and cell death. Occasionally, occurrence of smaller spheres was noted (Abram & Gibbons, 1961; Mohr & Larsen, 1963), and those derived from exposure to LiCl were deemed quite stable, but the phenomenon was not explored further.

### Dormant haloarchaea?

Of interest is the ATP content of spheres, which was about 50-fold lower than that of rods (Table 1). Data from dormant or starved bacteria and spores indicated similarly reduced amounts of ATP, for example, the ATP content of dormant spores of several *Bacillus* species was about two orders of magnitude lower than that of actively growing *Bacillus* cells (Setlow & Kornberg, 1970); marine *Vibrio* species, which are deemed dormant when in the oceans, lost ATP during starvation experiments (Oliver & Stringer, 1984); and several strains of *Staphylococcus* showed a decline of ATP when adhering to polymer surfaces and entering a dormant, miniaturized state (Stollenwerk *et al*., 1998). Prokaryotic dormancy is not easy to define precisely (Kell & Young, 2000), but generally, a low amount of cellular ATP has been associated with a dormant state of various bacteria and spores.

The intriguing findings by Schubert *et al*. (2009, 2010) of spherical particles of about the same size as those described here in fluid inclusions of ancient halite, which can be propagated to haloarchaeal cultures, are probably the best evidence so far for the occurrence of viable long-term survivors in geologically old sediments. Schubert *et al*. (2010) called the spherical particles miniaturized cocci; however, the haloarchaea that were grown from these particles – members of the genera *Halorubrum*, *Natronomonas* and *Haloterrigena* – were not halophilic cocci, but are known rod-shaped species (http://www.the-icsp.org/taxa/halobacterlist.htm). This suggested that the spherical particles in natural fluid inclusions might be in a dormant resting state, which is reversed to growth as rod-shaped or pleomorphic cells, once conditions for proliferation are met. Miniaturization of cells – sometimes called dwarfing (Nyström, 2004) – as consequence of starvation has been reported, besides *Staphylococcus* sp. (see above), for *Arthrobacter crystallopoietes* (Ensign, 1970), *Vibrio* sp. (Amy *et al*., 1983), and other bacteria (Nyström, 2004).

The data obtained in this work indicated a correlation of the formation of small spheres of halophilic archaea with reduced external water activity, which was brought about by high concentrations of NaCl or LiCl. The water activity of a saturated solution of NaCl at ambient temperature is 0.754 (http://www.emintech.com/1251exps.htm); sphere formation was never observed in such solutions, but rather in the fluid inclusion environment of halite. Lowering the a_w_ of a saturated NaCl solution could be achieved by the addition of other soluble salts, for example, Mg^++^ salts; these can lead, however, to denaturation of macromolecules and to cell death (Hallsworth *et al*., 2007). A buffered solution of 4 m LiCl with a_w_ of 0.73, on the other hand, was conducive for the formation of spheres and did not immediately impair the viability of haloarchaea. A detailed investigation of the mode of sphere formation and their properties should bring about insights how haloarchaea inside natural fluid inclusions, where low a_w_ is prevalent, may survive. Perhaps spheres can be identified as haloarchaeal resting states, for which no examples are known yet (Grant *et al*., 1998). Fluid inclusions were also found in billion-year-old meteorites and constitute apparently a very old type of structure in the universe (Zolensky *et al*., 1999). Thus, such studies may also be useful for the design of experiments aimed at the detection of potential extraterrestrial forms of microbial life in sediments of great geological age.

## SUPPORTING INFORMATION

**Fig. S1** SDS PAGE of soluble proteins from *Halobacterium salinarum* DSM 670 released upon exposure to Tris-buffered 4 m LiCl.

## References

[b1] Abram D, Gibbons NE (1961). The effect of chlorides of monovalent cations, urea, detergents, and heat on morphology and the turbidity of suspensions of red halophilic bacteria. Canadian Journal of Microbiology.

[b2] Adamski JC, Roberts JA, Goldstein RH (2006). Entrapment of bacteria in fluid inclusions in laboratory-grown halite. Astrobiology.

[b3] Amy PS, Pauling C, Morita RY (1983). Starvation-survival processes of a marine *Vibrio*. Applied and Environmental Microbiology.

[b4] Angert ER (2005). Alternatives to binary fission in bacteria. Nature Reviews Microbiology.

[b5] Arahal DR, Gutiérrez MC, Volcani BE, Ventosa A (2000). Taxonomic analysis of extremely halophilic archaea isolated from 56-years-old Dead Sea brine samples. Systematic and Applied Microbiology.

[b6] Breuert S, Allers T, Spohn G, Soppa J (2006). Regulated polyploidy in halophilic archaea. PLoS ONE.

[b7] Cline SW, Doolittle WF (1992). Transformation of members of the genus *Haloarcula* with shuttle vectors based on *Halobacterium halobium* and *Haloferax volcanii* plasmid replicons. Journal of Bacteriology.

[b8] Denner EBM, McGenity TJ, Busse H-J, Wanner G, Grant WD, Stan-Lotter H (1994). *Halococcus salifodinae* sp. nov., an archaeal isolate from an Austrian salt mine. International Journal of Systematic Bacteriology.

[b9] Dombrowski H (1963). Bacteria from Paleozoic salt deposits. Annals of the New York Academy of Sciences.

[b10] Ensign JC (1970). Long-term starvation survival of rod and spherical cells of *Arthrobacter crystallopoietes*. Journal of Bacteriology.

[b11] Erler A, Hawranek T, Krückemeier L, Asam C, Egger M, Ferreira F, Briza P (2011). Proteomic profiling of birch (*Betula verrucosa*) pollen extracts from different origins. Proteomics.

[b12] Fendrihan S, Stan-Lotter H, Teodorescu H, Griebel H (2004). Survival of halobacteria in fluid inclusions as a model of possible biotic survival in martian halite. Mars and Planetary Science and Technology.

[b13] Fendrihan S, Legat A, Pfaffenhuemer M, Gruber C, Weidler G, Gerbl F, Stan-Lotter H (2006). Extremely halophilic archaea and the issue of long-term microbial survival. Reviews in Environmental Science and Biotechnology.

[b14] Fish SA, Shepherd TJ, McGenity TJ, Grant WD (2002). Recovery of 16S ribosomal RNA gene fragments from ancient halite. Nature.

[b15] Gramain A, Chong Díaz GC, Demergasso C, Lowenstein TK, McGenity TJ (2011). Archaeal diversity along a subterranean salt core from the Salar Grande (Chile). Environmental Microbiology.

[b16] Grant WD, Gemmell RT, McGenity TJ (1998). Halobacteria – the evidence for longevity. Extremophiles.

[b17] Gruber C, Legat A, Pfaffenhuemer M, Radax C, Weidler G, Busse H-J, Stan-Lotter H (2004). *Halobacterium noricense* sp. nov., an archaeal isolate from a bore core of an alpine Permian salt deposit, classification of *Halobacterium* sp. NRC-1 as a strain of *H. salinarum* and emended description of *H. salinarum*. Extremophiles.

[b18] Hallsworth JE, Yakimov MM, Golyshin PN, Gillion JLM, D'Auria G, Alves FDL, La Cono V, Genovese M, McKew BA, Hayes SL, Harris G, Giuliano L, Timmis KN, McGenity TJ (2007). Limits of life in MgCl_2_-containing environments: chaotropicity defines the window. Environmental Microbiology.

[b19] Humble MW, King A, Philips I (1977). API Zym, a simple rapid system for the detection of bacterial enzymes. Journal of Clinical Pathology.

[b20] Kell DB, Young M (2000). Bacterial dormancy and culturability: the role of autocrine growth factors. Current Opinion in Microbiology.

[b21] Kiesslich T, Oberdanner CB, Krammer B, Plaetzer K (2003). Fast and reliable determination of intracellular ATP from cells cultured in 96 wells microplates. Journal of Biochemical and Biophysical Methods.

[b22] Kushner DJ, Bayley ST (1963). The effect of pH on surface structure and morphology of the extreme halophile *Halobacterium cutirubrum*. Canadian Journal of Microbiology.

[b23] Laemmli UK (1970). Cleavage of structural proteins during the assembly of the head of the bacteriophage T4. Nature.

[b24] Leuko S, Legat A, Fendrihan S, Stan-Lotter H (2004). Evaluation of the LIVE/DEAD BacLight kit for detection of extremophilic archaea and visualization of microorganisms in environmental hypersaline samples. Applied and Environmental Microbiology.

[b25] Lowry OH, Rosebrough NJ, Farr AL, Randall RJ (1951). Protein measurement with the Folin phenol reagent. The Journal of Biological Chemistry.

[b26] McGenity TJ, Gemmell RT, Grant WD, Stan-Lotter H (2000). Origins of halophilic microorganisms in ancient salt deposits. Environmental Microbiology.

[b27] Mescher MF, Strominger JL (1976). Structural (shape-maintaining) role of the cell surface glycoprotein of *Halobacterium salinarium*. Proceedings of the National Academy of Sciences of the USA.

[b28] Mohr V, Larsen H (1963). On the structural transformation and lysis of *Halobacterium salinarum* in hypotonic and isotonic solutions. Journal of General Microbiology.

[b29] Mormile MR, Biesen MA, Gutierrez MC, Ventosa A, Pavlovich JB, Onstott TC, Fredrickson JK (2003). Isolation of *Halobacterium salinarum* retrieved directly from halite brine inclusions. Environmental Microbiology.

[b30] Norton CF, Grant WD (1988). Survival of halobacteria within fluid inclusions in salt crystals. Journal of General Microbiology.

[b31] Norton CF, McGenity TJ, Grant WD (1993). Archaeal halophiles (halobacteria) from two British salt mines. Journal of General Microbiology.

[b32] Nyström T (2004). Stationary-phase physiology. Annual Review of Microbiology.

[b33] Oliver JD, Stringer WF (1984). Lipid composition of a psychrophilic marine *Vibrio* sp. during starvation-induced morphogenesis. Applied and Environmental Microbiology.

[b34] Onyenwoke RU, Brill JA, Farahi K, Wiegel J (2004). Sporulation genes in members of the low G+C Gram-type-positive phylogenetic branch (Firmicutes). Archives of Microbiology.

[b35] Park JS, Vreeland RH, Cho BC, Lowenstein TK, Timofeeff MN, Rosenzweig WD (2009). Haloarchaeal diversity in 23, 121 and 419 MYA salts. Geobiology.

[b36] Radax C, Gruber C, Stan-Lotter H (2001). Novel haloarchaeal 16S rRNA gene sequences from Alpine Permo-Triassic rock salt. Extremophiles.

[b37] Reiser R, Tasch P (1960). Investigation of the viability of osmophile bacteria of great geological age. Transactions of the Kansas Academy of Science.

[b38] Sára M, Sleytr UB (2000). S-layer proteins. Journal of Bacteriology.

[b39] Schubert BA, Lowenstein TK, Timofeeff MN (2009). Microscopic identification of prokaryotes in modern and ancient halite, Saline Valley and Death Valley, California. Astrobiology.

[b40] Schubert BA, Lowenstein TK, Timofeeff MN, Parker MA (2010). Halophilic Archaea cultured from ancient halite, Death Valley, California. Environmental Microbiology.

[b41] Setlow P, Kornberg A (1970). Biochemical studies of bacterial sporulation and germination. XXII. Energy metabolism in early stages of germination of *Bacillus megaterium* spores. The Journal of Biological Chemistry.

[b42] Smibert RM, Krieg NR, Gerhardt P, Murray RGE, Wood WA, Krieg NR (1994). Phenotypic characterization. Manual of Methods for General Microbiology.

[b43] Squyres S, Knoll AH (2005). Sedimentary rocks at Meridiani Planum: origin, diagenesis, and implications for life on Mars. Earth and Planetary Science Letters.

[b44] Squyres SW, Knoll AH, Arvidson RE, Clark BC, Grotzinger JP, Jolliff BL, McLennan SM, Tosca N, Bell JF, Calvin WM, Farrand WH, Glotch TD, Golombek MP, Herkenhoff KE, Johnson JR, Klingelhöfer G, McSween HY, Yen AS (2006). Two years at Meridiani Planum: results from the opportunity rover. Science.

[b45] Stan-Lotter H, McGenity TJ, Legat A, Denner EBM, Glaser K, Stetter KO, Wanner G (1999). Very similar strains of *Halococcus salifodinae* are found in geographically separated Permo-Triassic salt deposits. Microbiology.

[b46] Stan-Lotter H, Pfaffenhuemer M, Legat A, Busse H-J, Radax C, Gruber C (2002). *Halococcus dombrowskii* sp. nov., an archaeal isolate from a Permian alpine salt deposit. International Journal of Systematic and Evolutionary Microbiology.

[b47] Stan-Lotter H, Radax C, McGenity TJ, Legat A, Pfaffenhuemer M, Wieland H, Gruber C, Denner EBM, Ventosa A (2004). From intraterrestrials to extraterrestrials - viable haloarchaea in ancient salt deposits. Halophilic Microorganisms.

[b48] Stollenwerk M, Fallgren C, Lundberg F, Tegenfeldt JO, Montelius L, Ljungh A (1998). Quantitation of bacterial adhesion to polymer surfaces by bioluminescence. Zentralblatt für Bakteriologie.

[b49] Treiman AH, Gleason JD, Bogard DD (2000). The SNC meteorites are from Mars. Planetary and Space Science.

[b50] Vreeland RH, Jones J, Monson A, Rosenzweig WD, Lowenstein TK, Timofeeff M, Satterfield C, Cho BC, Park JS, Wallace A, Grant WD (2007). Isolation of live Cretaceous (121–112 million years old) halophilic *Archaea* from primary salt crystals. Geomicrobiology Journal.

[b51] Yang W, Spencer RJ, Krouse HR, Lowenstein TK, Casas E (1995). Stable isotopes of lake and fluid inclusion brines, Dabusun Lake, Qaidam Basin, western China: hydrology and paleoclimatology in arid environments. Palaeogeography, Palaeoclimatology, Palaeoecology.

[b52] Zolensky ME, Bodnar RJ, Gibson EK, Nyquist LE, Reese Y, Shih CY, Wiesman H (1999). Asteroidal water within fluid inclusion-bearing halite in an H5 chondrite, Monahans (1998). Science.

