# Supplementary figures and images for: Spherical particles of halophilic archaea correlate with exposure to low water activity – implications for microbial survival in fluid inclusions of ancient halite
Source: Geobiology. 2012 Jul 15;10(5):424–33. doi: 10.1111/j.1472-4669.2012.00337.x (PMC3495301; doi:10.1111/j.1472-4669.2012.00337.x)

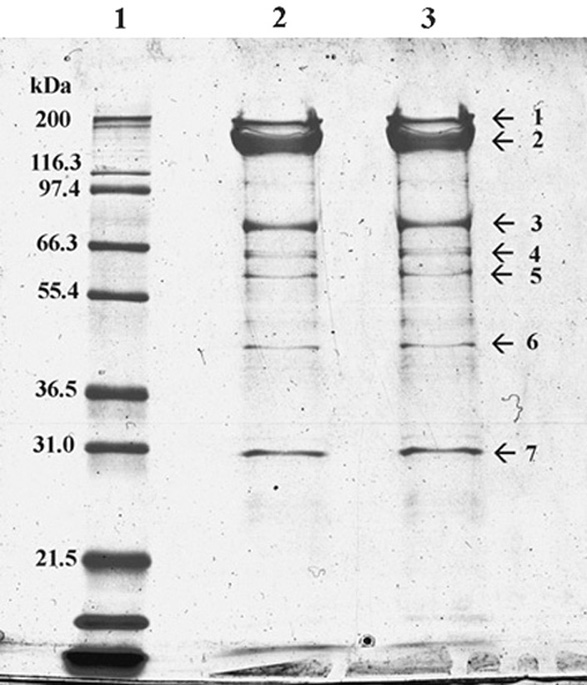

Supplement: Supplementary file 1 [file gbi0010-0424-SD1.tif]
